# Cell fusion in tumor progression: the isolation of cell fusion products by physical methods

**DOI:** 10.1186/1475-2867-11-32

**Published:** 2011-09-20

**Authors:** Filippo Pedrazzoli, Iraklis Chrysantzas, Luca Dezzani, Vittorio Rosti, Massimo Vincitorio, Giammaria Sitar

**Affiliations:** 1 Department of Internal Medicine IRCCS Policlinico San Matteo, viale Golgi 19, Pavia 27100 Italy and University of Pavia, Strada Nuova, Pavia 27100; Italy

## Abstract

**Background:**

Cell fusion induced by polyethylene glycol (PEG) is an efficient but poorly controlled procedure for obtaining somatic cell hybrids used in gene mapping, monoclonal antibody production, and tumour immunotherapy. Genetic selection techniques and fluorescent cell sorting are usually employed to isolate cell fusion products, but both procedures have several drawbacks.

**Results:**

Here we describe a simple improvement in PEG-mediated cell fusion that was obtained by modifying the standard single-step procedure. We found that the use of two PEG undertreatments obtains a better yield of cell fusion products than the standard method, and most of these products are bi- or trinucleated polykaryocytes. Fusion rate was quantified using fluorescent cell staining microscopy. We used this improved cell fusion and cell isolation method to compare giant cells obtained in vitro and giant cells obtained in vivo from patients with Hodgkin's disease and erythroleukemia.

**Conclusions:**

In the present study we show how to improve PEG-mediated cell fusion and that cell separation by velocity sedimentation offers a simple alternative for the efficient purification of cell fusion products and to investigate giant cell formation in tumor development.

## 1. Background

The study of cell fusion has gained momentum in modern cell biology through several lines of investigation [1-3]. Somatic cell hybrids have been used by a number of investigators to analyse the genetic basis of cancer [4-7] and for gene mapping [8-11]. Cell hybridisation provided the method for generating monoclonal antibodies [12] and fusion of virus-transformed cells has been instrumental in the isolation of transforming viruses [13]. Tumour-dendritic cell fusion technology is applied to immunotherapy strategies [14-16]. The idea that cell fusion plays a role in the transition from polyploidy to aneuploidy and hence in tumour progression has been the subject of several reviews [7,3], but experimental evidence in favour of this hypothesis is difficult to obtain. As noted by Brenda M. Ogle in a review on cell fusion, "the origin of a cell as the product of a fusion event can be difficult or impossible to deduce" [2].

In previous investigations we have explored the possibilities that lymphocyte-lymphocyte fusion plays a role in the cytogenesis of giant polykaryons in Hodgkin's disease (HD) [17] and that erythroblast fusion might be a source of giant polykaryons in erythroleukemia [18]. To further investigate these hypotheses, it might prove useful to isolate in-vitro-generated cell fusion products and compare giant cells obtained in vitro with those present in vivo, by morphological and immunocytochemical methods using anti- i monoclonal antibody. Poly-N-acetyllactosaminyl structures (i-antigen) carry a variety of physiologically and pathologically important carbohydrate antigens and are presumed to have essential roles in the process of cellular recognition, differentiation, malignant transformation and cancer metastasis[19].

Electrofusion [20] and PEG-induced chemical fusion [21] are the two most popular methods to fuse cells, and only recently have genetic methods been proposed [22]. PEG remains a widely used agent for cell fusion because of its simplicity and low cost. However, it is known that PEG can cause the uncontrollable fusion of multiple cells, leading to the appearance of giant polykaryons. In addition, standard PEG-mediated cell fusion is poorly reproducible, and different cell types have variable fusion susceptibilities. In an attempt to overcome these technical problems, we modified the classic one-step PEG fusion procedure by substituting two PEG undertreatments.

Genetic selection techniques [23] and fluorescent cell sorting [24,25] have been used to isolate cell fusion products, although both procedures have several drawbacks. Cell separation by physical methods provides a possible alternative when either the densities or radii of the cells to be isolated are substantially different in the starting cell fraction [26]. Because polykaryocytes generated by cell fusion are larger than their parent cells[27], it follows that velocity sedimentation should be an appropriate cell separation method. A relative large spherical particle, such as an animal cell, moving through an uniform stable medium under the influence of the earth's gravitational field, rapidly attains a terminal constant sedimentation that represent a balance between the gravitational force and the resistance to movement in a fluid. Equating the two forces, the terminal velocity V is equal to:

$$\text{V}=2\left(\rho_{\text{c}}-\rho_{\text{m}}\right)\text{g}{\text{r}}^{\text{2}}∕9\eta$$

where ρ_c _is cell density, ρ_m _is the density of the medium, r is the cell radius, η is viscosity of the medium and g is the earth's gravitational force. For most mammalian cells having densities in the range 1.040-1.090 g/ml, the difference in the term of ρ_c _- ρ_m _is usually small compared to the differences in radius, which is squared in equation: the sedimentation rate, therefore, is dependent primary on cell size. This suggests that separation by velocity sedimentation should be performed using a starting fraction that contains cells of restricted density.

Thus, the aim of this work is two-fold: 1) to provide a simple and efficient tool for obtaining cell fusion products and their isolation and 2) to offer preliminary evidence that cell fusion might be involved in giant cell formation in tumour pathology.

## 2. Materials and methods

### 2.1. Isolation of peripheral blood mononuclear cells

Peripheral blood cells were harvested from blood bank buffy coats, and mononuclear cells were isolated by density gradient centrifugation on lymphoprep (1.077 g/ml). Lymphocytes were further purified by overnight incubation in a tissue culture flask to deplete monocytes, which strongly adhere to the plastic. Erythroblasts were isolated from cord blood as previously described [28].

### 2.2. Cell labelling

For identification of fused cells, we divided the starting cell sample into two fractions that were labelled with either cell tracker green (CMFDA) or orange (CMTMR) (Molecular Probes, Eugene, OR) fluorescent probes according to the manufacturer's instructions. These dyes are fluorescent chloromethyl derivatives that freely diffuse through the membranes of live cells, but once inside the cell they are converted into membrane-impermeant reaction products, facilitating the identification of cell fusion products. CMFDA and CMTMR exchange minimally until the complete merger of two cells. Immediately after fusion the two dyes remain separate, but later a yellow fluorescence results from the mixing of orange and green dyes in fused cells. Additionally, in separated experiments, cells were labelled with Vydrant DiI (Molecular Probes, Eugene, OR), according to the manufacturer's instructions, in order to follow the sequence of events between the initial contact of cells and their fusion. Vybrant DiI it's a carbocyanine dye that labels cell membranes with an orange colour. Stained cells were continuously observed in phase contrast/fluorescent microscopy to monitor cell fusion.

### 2.3. PEG-induced cell fusion

PEG-mediated cell fusion was performed either in a single step procedure or in two steps over an interval of 15 h. Cells previously labelled with fluorescent probes were treated with 1 ml 35% PEG 6000 MW (Merck-Schuchardt, Hohenbrunn, Germany) in RPMI 1640 medium (Sigma, St. Louis, MO), pH 8.2, for an exposure time of either 3 or 5 or 8 minutes (single step procedure) in a water bath at 37°C. During this time, the tube was gently swirled to keep cells in suspension.

10 ml MEM (Minimal Essential Medium, Sigma, St. Louis, MO) containing DNase was added in 10 mins by continuous flow through a peristaltic pump. DNase was added to prevent cell clumping. Cells were then centrifuged to remove PEG, washed twice in RPMI 1640, and resuspended in complete medium (RPMI 1640 + 10% fetal calf serum + 2 mM glutamine, penicillin, and streptomycin). For the two steps procedure, labeled cells were initially treated for 3 minuts with with 1 ml 35% PEG 6000 MW in a water bath at 37°C using the same protocol. After overnight incubation at 37°C/5% CO_2_, PEG treatment was repeated for 5 min using the same protocol.

After each PEG treatment, cell fusion products were evaluated and counted by cytochemistry and phase contrast/fluorescence microscopy. Cell viability was determined by trypan blue exclusion. Photographic documentation was obtained using Zeiss fluorescence microscope and a Leica confocal microscope TCS SP2.

### 2.4. Cell co-culture

Cell culture was performed in Transwell 6-well co-culture chambers (Costar, Cambridge, MA) as previously described [29]. These chambers were designed so that the lower compartment, where a first cell population is placed in culture, is separated from the upper compartment by a 0.45-μm microporous membrane permeable only to viruses and cytokines. In the lower compartment, peripheral blood mononuclear cells (PBMC) from HD patients were seeded, while the upper compartment contained either an autologous single-cell suspension from a HD [30] lymph node obtained for diagnostic purposes or an Epstein Barr virus producing cell line (Burkitt's lymphoma derived line P_3_HR-1, Human Tumor cell Bank-HTB).

### 2.5. Separation of cell fusion products by velocity sedimentation

**Figure 1:**
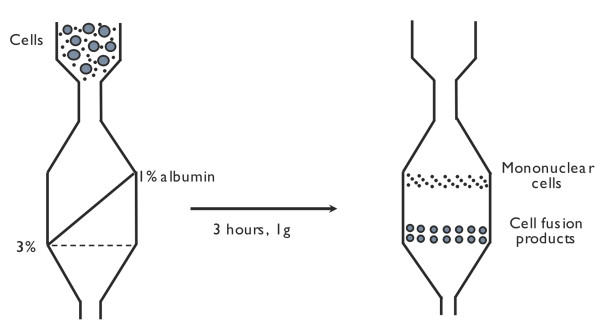
**Isolation of cell fusion products by velocity sedimentation**. After three hours a clear-cut separation is obtained between polykaryocytes and mononuclear cells.

For cell separation by velocity sedimentation, we used a sedimentation chamber previously described for the isolation of Reed-Sternberg cells [31]. The separation chamber was filled from the bottom with a linear gradient (1% - 3%) of human albumin in RPMI, generated with a gradient mixer and peristaltic pump. The gradient was underlayered with a dense liquid, immiscible with water (Fluorinert, 3M Co, St Paul, MN) that brought the gradient up to the top of the sedimentation chamber (Figure 1).

Next, the cell sample was introduced into the chamber and onto the gradient by reversing the peristaltic pump, thus lowering the cell sample to the cylindrical part of the device. This procedure deposits a very thin band (less than 1 mm) of an undisturbed cell suspension onto the gradient. Cells were allowed to sediment at unity gravity for 3 h, and then sequential fractions of 25 ml each were collected from the bottom of the sedimentation chamber. The percentages of mono-, bi-/tri-, and polynucleated cells in each fraction were evaluated by cytochemistry and fluorescence microscopy.

### 2.6 Culture in vitro of isolated cell fusion products and immunofluorescent labelling

Following separation by velocity sedimentation, fused cells were incubated at 37°C in tissue culture medium. Samples were seeded in a classical clonogenic assay in semisolid medium. Briefly, 2 × 10^4 ^cells were plated in 30 mm Petri dishes in 1 ml aliquot of IMDM in the presence of 30% FCS, 10 ng/ml IL-3, and 0.9% methylcellulose (w/v). After 4 weeks colonies were located under an inverted microscope, individually picked up, washed and rendered into a single cell suspension before being cytocentrifuged. Immunostaining with anti-i was performed as previously described [32]. Slides were incubated overnight, at 4°C, with monoclonal antibody anti-i diluted 1:200 followed by incubation with secondary monoclonal anti-IgM-TRITC. Nuclei were counterstained by DAPI (4',6-diamini-dino-2-phenylindole) for 5 minute at room temperature.

## 3. Results

### 3.1. Cell fusion

Table 1 shows fusion rates for a homogeneous sample of lymphocytes using different times of PEG treatment. Experiments with a single PEG exposure showed poor fusion efficiency when a short fusion time was used. When the fusion time was increased, large sincytias with many nuclei appeared and cell viability substantially decreased. Differently when a two-steps PEG undertreatment procedure was used, the fusion efficiency improved as shown in Table 2. The highest rate of fusion was obtained using a homogeneous population of lymphocytes, while erythroblasts had intermediate fusion ability.

**Table 1:** Cell fusion after a single PEG treatment using three different times of PEG exposure: mean ± standard deviation.

Time of PEG-exposition	Cell sample Lymphocytes (× 10^6^)	Survival (%)	Cell fusion products (%)	Bi-trinucleated cells (%)	Polynucleated cells (5-8 nuclei) (%)
**3 min**.	94.22 ± 5.4	77.2 ± 1.98	1.93 ± 0.08	1.87 ± 0.13	0.22 ± 0.42
**5 min**.	94.7 ± 6.15	47 ± 4.06	5.84 ± 1.51	2.93 ± 0.85	2.94 ± 0.82
**8 min**.	93.64 ± 4.87	21.9 ±1.95	12.06 ± 4.68	2.9 ± 0.52	8.88 ± 4.1

1000 cells were counted in phase contrast microscopy (data from 10 experiments).

**Table 2:** Fusion of homologous and non-homologous cells: mean **± **standard deviation.

Cell sample	Number of cells (× 10^6^)	Bi-tri nucleated cells (%)	Poly nucleated cells (%)	Single-dye polykaryocytes (%)	Dual-dyes polykaryocytes (%)
**Ly-Ly** **1^st ^step**	98 ± 3.4	1.3 ± 0.8	0.11 ± 0.22	1.44 ± 0.24	0.35 ± 0.04
**Ly-Ly** **2^nd ^step**	17.9 ± 0.6	79.61 ± 0.86	8.9 ± 5.2	9.5 ± 1.2	7.73 ± 2.5
**Er-Er** **1^st ^step**	1 ± 0.06	0.76 ± 0.01	1.04 ± 0.8	0.74 ± 0.05	0.33 ± 0.66
**Er-Er** **2^nd ^step**	7.02 ± 0.11	3.3 ± 0.5	2.9 ± 0.95	2.8 ± 1.09	1.44 ± 0.87
**Ly-Mo** **1^st ^step**	99 ± 0.91	0.48 ± 0.05	0.24 ± 0.01	0.25 ± 0.09	0.22 ± 0.03
**Ly-Mo** **2^nd ^step**	70.07 ± 1.85	2.23 ± 0.02	1.75 ± 0.84	1.63 ± 0.78	0.71 ± 0.06

1000 cells were counted in phase contrast microscopy and in fluorescent microscopy using a filter set appropriate for both emission wavelenghts (CMFDA and CMTMR). (Data from 10 experiments. Ly, lymphocyte; Er, erythroblast; Mo, monocyte.). Single-dye polykaryocytes result from the fusion of cells stained by the same fluorescent dye, while dual-dye polykaryocytes result from the fusion of cells stained bt two different fluorescent dyes.

An unexpected result using this approach is that most fusion products were bi- or tri-nucleated cells. Giant multinucleated cells (5-10 nuclei) were consistently less than 2% of the cell population after the first PEG undertreatment step. Microscopic observation of cell fusion after the two-step procedure showed that 16-24% of lymphocytes were physically fused. A mean number of 1,000 cells were counted using phase contrast/fluorescent microscopy.

### 3.2. Microscopic examination of fused cells

**Figure 2:**
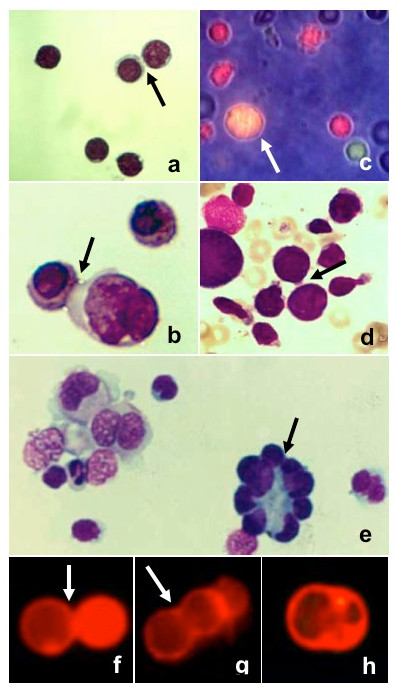
**Cells after the first PEG undertreatment (May-Grünwald Giemsa staining)**. Intercellular bridges (black arrows) were clearly visible: (a) lymphocytes (400×), (b) erythroblasts (800×). One fusion product (white arrow) was present (c) among single cells (green and red), as observed by fluorescent and phase contrast microscopy (500×). (d) Intercellular bridges between erythroblasts (arrow) in a bone marrow smear from a case of erythroleukemia (500×). (e) Single cell suspension from a lymph node affected by Hodgkin's disease (500×). (f) Membrane apposition, (g) triggering of fusion, (h) membrane coalescence, in cells stained by Vybrant DiI (500×).

After the first PEG undertreatment, we observed in cytocentrifuged preparations several cells in close contact, sometimes connected by microspikes to touch and adhere to adjoining cells (Figure 2a-b), as previously described by others [33], but few fusion products (Figure 2c). The second morphological feature of early fusion are intercellular bridges closely resembling similar morphological structures we observed in bone marrow cells from an erythroleukemia patient [18], suggesting cell fusion (Figure 2d) and in single cell suspension from lymph node affected by Hodgkin's disease (Figure 2e).

**Figure 3:**
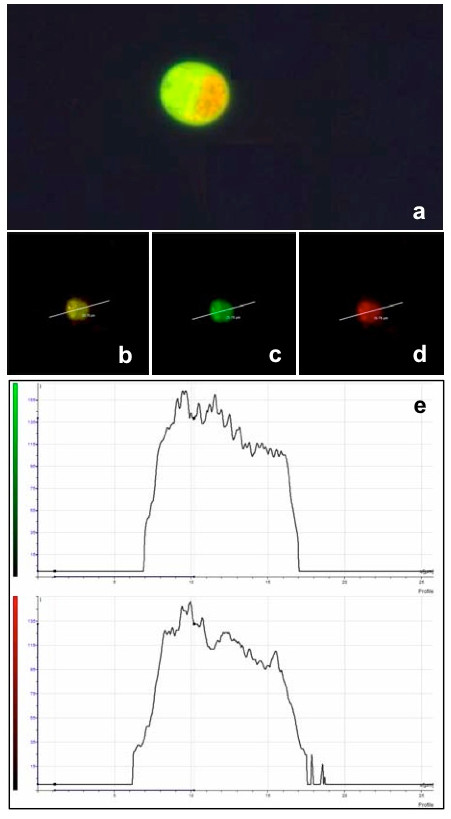
**A representative image of a cell fusion product, lymphocyte-lymphocyte, (a) observed in fluorescence microscopy (800×), using a dual band filter for both emission wavelenghts of CMTMR and CMFDA**. This same cell type was observed using a Leica TCS SP2 confocal microscope. The green fluorescent emission was recorded between 500/530 nm (c) and the red one between 570/620 nm (d). The colocalization of the two colours in the fusion product is evidenced by the emission of both the green and red fluorescence at the same plane (b and e).

To further characterize the sequence of events following the initial contact of cells, we monitored continuously the morphological changes during fusion of individual cell. Cells, stained by Vybrant DiI, made initial contact (Figure 2f) and didn't experience observable morphological changes during several minutes. Then cells enlarged slightly and the boundary between two cells began to disappear (figure 2g) and was completed within two-three minutes leading to rounding of the fusion product (figure 3h).

A representative example of the fusion product of two individual cells, one stained by CMFDA and the other by CMTMR, is shown in Figure 3a. The two dyes produce a yellow colour only when there's a balanced quantity of the two dyes and when viscosity of the cytoplasm allows a balanced mixing of the dyes. This same cell type was observed by confocal microscopy using different excitation/recording laser lines appropriate for the two dyes (Figure 3b-d).

**Figure 4:**
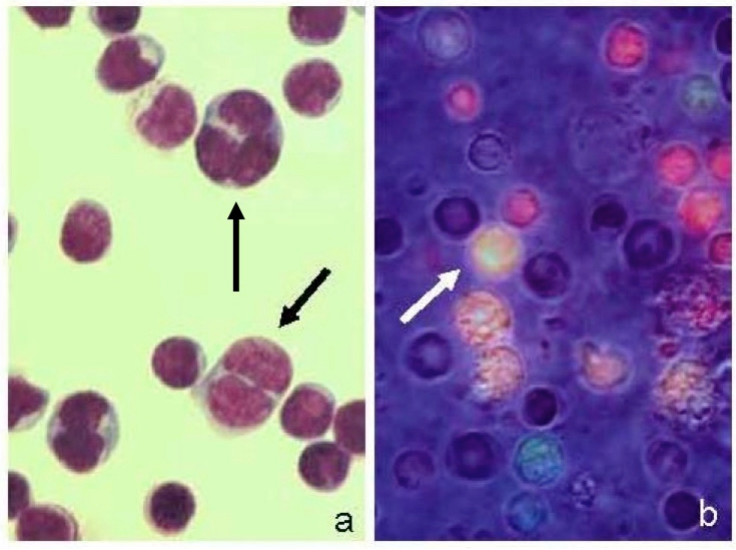
**Lymphocytes after the two-step fusion procedure and before separation by velocity sedimentation**. (a) Cells were cytocentrifuged on a glass slide and stained by May-Grünwald Giemsa. Bi- and trinucleated cells (black arrows) were readily observed (800×). (b) Cell fusion products, in vivo, were transferred into an Iwaki quartz base dish and observed by inverted fluorescence microscopy using a dual filter (485/515 nm and 578/610 nm) and by phase contrast microscopy (500×). One fusion product, at the bottom, is entirely green (two CMTMR-labeled cells fused) while the three fusion products (white arrow) in the centre-left are yellow (one CMTMR-labeled cell fused with a CMFDA-labeled cell). The mixing of the two fluorescent dyes takes some time and it is mainly dependent on temperature and the viscosity of cell cytoplasm. Yellow fluorescent is evident only when the two fluorescent dyes, CMTMR and CMFDA, are completely mixed.

**Figure 5:**
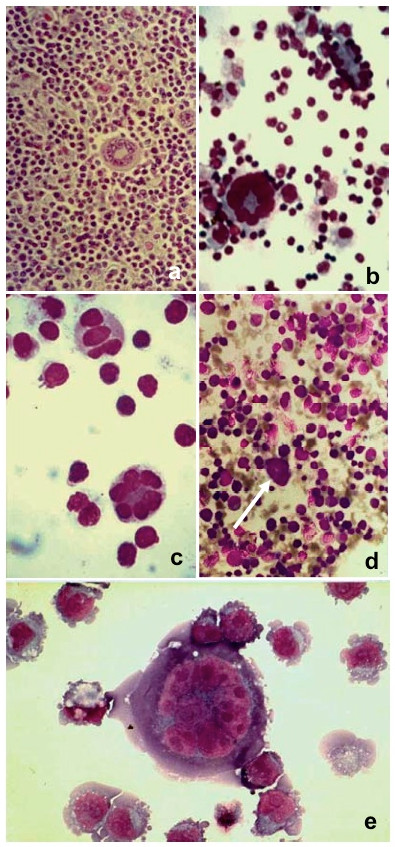
**(a) Histological section of a lymph node from a case of mixed cellularity HD**. A Reed-Sternberg cell with nuclei arranged in a circular fashion at the cell periphery is observed in the centre of the picture (200×). (b) Giant polykaryon obtained in vitro when peripheral blood mononuclear cells (PBMC) from an HD patient were co-cultivated with a single cell suspension from the same patient in a Transwell co-culture chamber. The two cell samples are separated by a 0.4-μm microporous membrane permeable to viruses and cytokines. At lower left is a giant polykaryon, and at upper right are several cells in the process of fusing (400×). (c) Giant polykaryon observed after two-step PEG treatment (800×). (d) Giant polykaryon (white arrow) in a bone marrow smear from a case of erythroleukemia (200×). (e) Giant polykaryon from PBMC co-cultivated with P_3_HR-1 subline in a Transwell co-culture chamber (800×).

After the second PEG undertreatment many cell fusion products were readily observed (Figure 4a-b). Most cell fusion products were bi-trinucleated homokaryocytes or heterokaryocytes while a small percentage were giant polykaryons, some with nuclei arranged along the periphery of the cell (Figure 5c). Wreath-like giant cells can be frequently observed in HD lymph nodes (Figure 5a) and we have obtained morphological similar cells when PBMC were co-cultivated either with an autologous single-cell suspension of HD lymph node (Figure 5b) or with the P_3_HR-1 cell line (Figure 5e), in a Transwell co-culture chamber where the two cell populations are separated by a 0.45-μm microporous membrane. In Figure 5b arrow shows several cells in the process of fusing, close to a giant polykaryon, morphologically resembling a cytological pattern observed in Figure 1e. If this morphological pattern represents sequential steps of a fusion process is presently suggestive, but remains to be proved. Similar cells were also observed in erythroleukemia bone marrow (Figure 5d).

### 3.3. Isolation of cell fusion products

**Figure 6:**
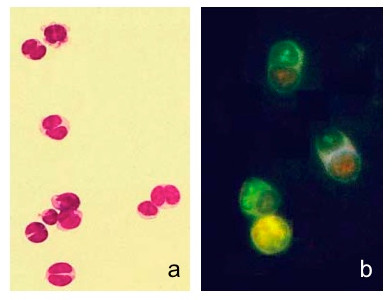
**(a) Cell fusion products isolated by velocity sedimentation (fraction III) and stained by May-Grünwald Giemsa (400×) after cytocentrifugation, and (b) observed, in vivo, by fluorescence microscopy (500×) using a dual filter (485/515 nm and 578/610 nm) Cell tracker green (CMFDA) and orange (CMTMR) can still be observed after fusion; a yellow fluorescence develops only after cytoplasmic mixing**.

Fused cells were isolated by velocity sedimentation. After 3 h of sedimentation, cell fusion products were recovered in the fast-moving fractions (Table 3). Polykaryons with several nuclei (4-6) were almost exclusively obtained in the first fraction, while later fractions contained mostly bi- or trinucleated cells. The mononuclear unfused cell fraction moved at the sedimentation velocity of 6 mm/h, and a clear-cut separation between the slow moving and fast moving cell fractions was always obtained. The accuracy of the enrichment procedure was evaluated by counting a mean number of 1,000 cells per sample (Figure 6a-b).

**Table 3:** Isolation of lymphocyte-lymphocyte fusion products by velocity sedimentation, mean **± **standard deviation.

Cell isolation by velocity sedimentation		Mononucleated cells (%)	Bi-tri nucleated cells (%)	Polynucleated cells (%)
**Fractions**	**I**	0.44 ± 0.75	0.48 ± 0.63	98.88 ± 1.01
	**II**	0.21 ± 0.20	5.09 ± 0.87	95.97 ± 0.67
	**III**	3.81 ± 0.83	95.94 ± 0.82	0.33 ± 0.72
	**IV**	87.06 ± 0.85	13.16 ± 0.85	0.2 ± 0.11

1000 cells were counted in phase contrast microscopy and in fluorescent microscopy using a filter set appropiate for both emission wavelenghts (CMFDA and CMTMR). (Data from 10 experiments.)

### 3.4 Immunostaining of cell fusion products

**Figure 7:**
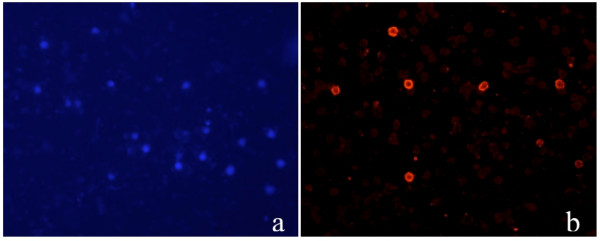
**Staining for i-Ag of cells retrieved from in vitro culture of cell fusion products after PEG-induced cell fusion of lymphocytes**. a. DAPi staining of nuclei b. Not all cells are i-Ag positive when stained with anti-i followed by incubation with secondary monoclonal anti-IgM-TRITC

After in vitro culture of cell fusion products obtained from lymphocytes isolated from adult peripheral blood, the majority of colonies were i-Ag negative while few of them were positive when stained with anti-i monoclonal antibodies (Figure 7a-b), the proportion of i-Ag positive cells didn't change with longer in vitro culture.

## 4. Discussion

The frequency of cell-cell fusion events induced by PEG using standard conditions is usually low, and this quantitative limitation can impede subsequent investigations. Therefore, we wished to develop an improved method for PEG-mediated cell fusion that yields a higher number of cell fusion products.

PEG damages cells. Consequently, the success of fusion depends at least to some extent on minute details such as the size and shape of the cell pellet, the intensity of shaking, and other factors that are difficult to standardize. PEG's effect on cells is mainly secondary to dehydration forces bringing the cells together. However, at least at a lipid interface, PEG changes the orientation and configuration of molecular dipoles and the organization of water molecules associated with the membrane. Thus, PEG probably induces changes in cell membrane permeability and in the properties of its cytoskeletal components, which may explain the cell rounding observed during fusion. PEG overtreatment results in excessive cell death and the formation of sincytia containing several nuclei, while undertreatment results in insufficient fusion.

Our strategy to improve PEG-induced cell fusion was suggested by the two-step electrofusion technique, which first accomplishes cell membrane breakdown by a high-intensity direct current pulse, followed by a second pulse that induces cell fusion. Similarly, we adopted a two-step approach to PEG-induced cell fusion. Using two under treatments with 35% PEG for 3 min instead of the standard single 50% PEG treatment for 5 min, we were able to obtain a high yield of cell fusion products. Importantly, most of these fusion products were bi- or trinucleated cells.

Once we had devised a method to improve PEG-mediated cell fusion, we addressed the problem of isolating cell fusion products. Genetic selection techniques such as the HAT (hypoxanthine, aminopterin, thymidine) system have been employed to isolate cell fusion products [12], although there are several practical and theoretical problems associated with their use. For example, only special cell lines that lack the required enzymes can be used. A second limitation is that substantial bias is introduced by the fact that most genetic selection systems depend on the ability of the fusion product to divide in selective medium. The inability to utilize genetic selection techniques for normal cells represents a limitation for many studies, such as the production of immunovaccines for tumour immunotherapy by dendritic-tumour cell fusion [34-36].

Flow cytometry has been offered as an alternative approach to quantifying and isolating hybrid cells after fusion. Dot plot-type fluorescence-activated cell sorting shows a quadrant in which a fluorescently coloured probe monitors one cell type, and a second coloured probe tracks a second cell type. A dually staining "fusion" quadrant is considered demonstrative of fusion. However, this evidence is not conclusive because aggregated cells and single cells coated with cell debris can appear in this "fusion" quadrant. Indeed, confocal microscopy confirmed these difficulties and proved to be superior to flow cytometry in detecting cell hybrids [25].

On the basis of these theoretical considerations, it seemed useful to isolate polykaryocytes, after PEG fusion, by procedures independent from genetic selection or fluorescent tracking. Cell separation by physical methods is an alternative approach to isolating these polykaryocytes.

The theory underlying cell sedimentation holds that the rate of sedimentation is largely a function of cell size, suggesting velocity sedimentation as an appropriate method for purifying cell fusion products [27]. Accordingly, we found that separation of bi- and trinucleated cells was excellent due to their larger size than parent cells.

During this investigation, we made the accidental observation of some similar morphological features between in-vitro-fused cells and polykaryons we previously observed in HD [29] and erythroleukemia [18]. Intercellular bridges were frequently observed after the first step of PEG undertreatment (Figure 1a-b) that closely resembles analogous structures present in a bone marrow preparation from an erythroleukemia patient (Figure 1d). Furthermore, large multinucleated cells with nuclei arranged in a circular fashion along the cell periphery can be observed both in vivo (in erythroleukemia and HD) and in vitro under different experimental cell fusion conditions (Figure 4). Jin has described similar cells in experiments when human kidney epithelial cells were exposed to chronic activation of protein kinase B [37] and by McShane while investigating EBV-induced cell fusion [38].

Hodgkin and Reed-Sternberg cells (HRS cells) are believed to originate from B cells, but their phenotypic pattern is very heterogeneous. B lineage markers such as CD20, B-cell receptor, and CD79a are found only rarely on these cells, and the lack of surface immunoglobulin expression sharply distinguishes them from their healthy counterparts. Whether this results from a) mutations within the rearranged immunoglobulin genes or non-functional rearrangements that prevent expression of these genes [39], or b) the extinction phenomenon that takes place when two cells fuse [40,41]., remains an unanswered question.

Overall, the peculiar morphology and immunophenotype of HRS cells in infectious mononucleosis, HD, and other diseases are still a mystery. If cell fusion plays a role in giant cell formation in HD is presently only a tempting hypothesis that is difficult to prove [2]. In infectious mononucleosis and in several cases of HD, Reed-Sternberg cells can be linked to infection with Epstein-Barr virus, a fusogenic agent. Stepwise transformation of virus-infected cells, possibly triggered by inherent genetic instability, might lead to aneuploidy and cancer [42]. The correlation between aneuploidy and cancer has been known for decades; however, the central question of whether aneuploidy results from cell fusion or abortive mitosis is still unanswered.

Altered expression pattern for the I/i antigens have often been observed during oncogenetic processes as well as in benign haemopoietic disorders [43] Poly-N-acetyllactosaminyl, I (branched structure) and i (linear structure) structures, are important carbohydrate antigens and are presumed to have essential roles in the process of cellular recognition, differentiation [44], malignant transformation and cancer metastasis, they are also expressed in carcinoma cells in several tissues and organs [45]. The i/I transition in cell differentiation is determined by the transcription factor CCAAT/enhancer binding protein alpha (C/EBPalpha), which enhances transcription of the IGnTC gene, consequently leading to formation of the I antigen. The loss of function of C/EBPalpha has leukemogenic potential by remodelling the transcription network of B cells through a series of parallel and sequential changes that require endogenous PU.1 [46].

With this in mind we investigated the i/I antigen expression in cell fusion products of lymphocytes isolated from peripheral blood and kept in vitro culture for prolonged time. The presence of i-antigen positive colonies suggest that cell fusion interferes with normal differentiation and gene expression. Microarray technology is a powerful tool that can quantify the expression of thousands of genes in a single analysis. It has the potential to monitor chromosome gains and losses, accomplish DNA resequencing, and detect mutations, allowing more intense probing of the mechanisms of tumour development. A "census of human cancer genes" was compiled that lists selected cancer genes having a causal link to mutation and oncogenesis [47]. Using a microarray approach to compare Reed-Sternberg cells obtained in vivo and similar cells obtained in vitro might contribute to unravelling the enigma if cell fusion plays a role in Reed-Sternberg cell cytogenesis.

In conclusion, we have introduced some technical improvements in PEG-mediated cell fusion and in the isolation of cell fusion products that might prove useful in the study of human malignancy, in the field of gene mapping, and we have offered preliminary evidence that cell fusion might be involved in giant cell formation in HD.

## Competing interests

The authors declare that they have no competing interests.

## Authors' contributions

FP carried out cell fusion experiments and participated in the design of the study, IK and LD carried out cell culture in vitro and immunofluorescent investigation, VR participated in its design and coordination and helped to draft the manuscript, GMS designed the study and drafted the manuscript. All authors read and approved the final manuscript.
